# Editorial: Nutrients, stress response, and human health

**DOI:** 10.3389/fnut.2025.1558682

**Published:** 2025-02-27

**Authors:** Jagannath Misra, Julius Liobikas

**Affiliations:** ^1^Department of Biochemistry and Molecular Biology, Indiana University School of Medicine, Indianapolis, IN, United States; ^2^Laboratory of Biochemistry, Neuroscience Institute, Medical Academy, Lithuanian University of Health Sciences, Kaunas, Lithuania

**Keywords:** nutritional metabolites, oxidative stress, cardiometabolic health, aging and longevity, metabolic dysfunction, gene-diet interactions, stress response pathways

Although significant progress has been made in understanding nutrient signaling pathways and their role in cellular health, the complexity of these mechanisms has revealed that their impact extends far beyond basic metabolic functions. Nutrient availability and sensing are now recognized as critical determinants of cellular adaptation to stress, with far-reaching implications for human health. The interplay between nutrient sensing, stress response pathways, and metabolic regulation holds the key to addressing conditions ranging from cancer progression to metabolic disorders. This Research Topic, titled “*Nutrients, Stress Response, and Human Health*” brings together contributions that explore the intricate dynamics of nutrient signaling in cellular physiology. Each study sheds light on distinct yet interconnected aspects of how nutrient homeostasis influences health and disease, providing a comprehensive view of this essential biological theme.

Wang et al. explore the causal relationship between circulating serum metabolites (CSMs) and hemorrhagic stroke (HS) through rigorous Mendelian randomization analysis. Their findings identify specific metabolites, such as biliverdin and linoleate, as protective factors for intracerebral hemorrhage (ICH), while others like 1-eicosadienoylglycerophosphocholine increase the risk of subarachnoid hemorrhage (SAH). These results emphasize the role of metabolites in influencing inflammation, oxidative stress, and lipid homeostasis, thus uncovering actionable biomarkers and pathways relevant to stroke prevention. This study lays a foundational understanding of how metabolic health can be influenced by circulating biomarkers.

Expanding on the theme of metabolic influences, Yu et al. investigate the relationship between the triglyceride-glucose (TyG) index and the risk of aortic aneurysm and dissection (AAD). They highlight the predictive power of TyG-related indices, particularly TyG-waist circumference (TyG-WC), in identifying high-risk individuals. This study underscores the role of metabolic health markers in cardiovascular disease risk stratification, complementing Wang et al.'s focus on metabolic pathways by emphasizing the translational value of early detection metrics in clinical practice.

The role of dietary interventions in modulating metabolic pathways is explored by Liu et al., who studies the nutritional regulation of aging, examining the relationship between folate intake and serum Klotho levels in adults. This cross-sectional study identifies folate as a modulator of Klotho, a protein implicated in aging-related diseases and longevity. The findings highlight the role of specific nutrients in regulating aging pathways, suggesting that folate may help mitigate age-related diseases. This research emphasizes the importance of nutrient-stress response interactions in promoting healthy aging, resonating with the findings of Wang et al. and Yu et al. by highlighting the significance of dietary components in systemic health regulation.

Adding to the dietary narrative, Roumi et al. investigate the interplay between polyphenol intake, genetic predispositions, and cardiometabolic risk factors in overweight and obese women. Their study demonstrates significant gene-diet interactions, with specific polyphenol types influencing markers like HDL cholesterol and triglycerides. These findings suggest the potential for personalized nutrition strategies, aligning with Liu et al.'s emphasis on tailored dietary interventions to mitigate metabolic risks.

Odetayo et al. address nutrient-driven modulation of oxidative stress and inflammation in their investigation of omega-3 fatty acids (O3FA) as protective agents against tamoxifen-induced gonadotoxicity. Their results highlight O3FA's ability to restore redox balance, suppress inflammatory pathways, and mitigate apoptosis, offering mechanistic insights into how dietary supplements can counteract drug-induced side effects. This study builds on the oxidative stress narratives introduced by Wang et al. and Roumi et al., emphasizing the therapeutic potential of nutrients in stress response pathways.

Continuing with oxidative balance, Yuan et al. explore the association between oxidative balance score (OBS) and serum cobalt levels in individuals with metal implants. Their findings reveal an inverse relationship, particularly in older males, and propose antioxidant-rich diets as strategies to mitigate implant-related oxidative stress. This study's emphasis on dietary antioxidants complements Odetayo et al.'s findings, reinforcing the role of oxidative stress modulation in health maintenance.

Finally, Amer et al. investigate the metabolic effects of glucose supplementation in high-fat diet mouse models, shedding light on the dual role of glucose in accelerating liver injury and promoting lipid oxidation. Their findings provide a critical perspective on how excessive dietary components can exacerbate metabolic dysfunction. While certain nutrient components exhibit clear protective effects on human health, as highlighted by Liu et al.'s findings on dietary folate and its positive role in enhancing serum Klotho levels, and Roumi et al.'s demonstration of polyphenols mitigating cardiometabolic risks, it is equally critical to recognize the potential harms associated with excessive nutrient intake. Amer et al.'s work underscores this cautionary principle by illustrating how excessive glucose supplementation, particularly with L-glucose, exacerbates liver injury in mice. These findings collectively offer a balanced view of nutrient impacts on metabolic health.

## Conclusion

In conclusion, these articles collectively illuminate the intricate connections between nutrients, stress responses, and human health. They underscore the importance of metabolic markers, dietary components, and oxidative stress modulation in understanding disease mechanisms and developing preventive strategies. This Research Topic is both timely and crucial, given the rising global burden of metabolic and age-related disorders. Together, these studies provide a robust foundation for advancing our understanding of how targeted dietary and metabolic interventions can improve health outcomes, offering actionable insights for both clinical and public health applications.

